# High resolution crystal structure data of human plasma retinol-binding protein (RBP4) bound to retinol and fatty acids

**DOI:** 10.1016/j.dib.2018.03.112

**Published:** 2018-03-29

**Authors:** Massimiliano Perduca, Stefania Nicolis, Barbara Mannucci, Monica Galliano, Hugo L. Monaco

**Affiliations:** a Biocrystallography Laboratory, Department of Biotechnology, University of Verona, Ca Vignal 1, strada Le Grazie 15, 37134 Verona, Italy; b Department of Chemistry, University of Pavia, via Torquato Taramelli 12, Pavia, Italy; c Centro Grandi Strumenti (CGS), University of Pavia, Agostino Bassi 21, Pavia, Italy; d Department of Molecular Medicine, University of Pavia, via Taramelli 3b, 27100 Pavia, Italy

## Abstract

Retinol is transported in vertebrate plasma bound to a protein called retinol-binding protein (RBP4) so far believed to be specific for the vitamin. When the protein is saturated with retinol it binds tightly to another plasma protein, transthyretin while when not saturated with retinol it does not bind to TTR (Goodman, 1984). The X-ray structures of human RBP4, holo and devoid of retinol in its binding site are known to resolutions of 2.0 and 2.5 Å (Cowan et al., 1990; Zanotti et al., 1993) [2], [3]. We have shown that RBP4 is not specific for retinol but it is also found in plasma, urine and amniotic fluid bound to fatty acids. Here we present 1.5 Å resolution crystal data on human plasma retinol-binding protein bound to retinol and fatty acids. These are the highest resolution data available in the Protein Data Bank for this protein.

For further details and experimental findings please refer to the article “ Human plasma retinol-binding protein (RBP4) is also a fatty acid-binding protein” (Perduca et al., 2018) [4].

**Specifications Table**

**Table:** 

Subject area	*Biology*
More specific subject area	*Structural Biology*
Type of data	*Single crystal x-ray diffraction*
How data was acquired	*ESRF European Synchrotron radiation Facility*
Data format	*Standard, required by the PDB*
Experimental factors	*Structure Factors*
Experimental features	*Structures solved by molecular replacement*
Data source location	*Grenoble, France*
Data accessibility	*Available in the Protein Data Bank. Accession codes listed below*

**Value of the data**.

The extension of the resolution limit of RBP4 permitted the removal of several ambiguities in the models of the holo protein available at lower resolution [2], [3]. More important was the identification of a heretofore unidentified ligand in the binding site of the protein believed to be apo. This second result changes substantially our perception of this protein that has so far been considered to be specific for retinol [1].

## 1 Data

The data of this article provides information on the X-ray crystallographic data sets of RBP4 purified from different sources. All these data are accessible at the Protein Data Bank. The table below summarizes origin, crystal resolution and PDB accession codes.

**Table:** 

**Source of RBP4 crystal data set**	**Resolution**	***PDB Accession Code***
RBP4 not bound to retinol purified from plasma	2.0 Å	5NTY
RBP4 not bound to retinol purified from urine	1.5 Å	5NU2
RBP4 not bound to retinol purified from amniotic fluid	1.7 Å	5NU6
Plasma Holo RBP4 (retinol)	1.5 Å	5NU7
Urine RBP4 saturated with palmitate	1.6 Å	5NU8
Amniotic Fluid RBP4 saturated with palmitate	1.5 Å	5NU9
Urine RBP4 saturated with laurate	1.6 Å	5NUA
Amniotic Fluid RBP4 saturated with laurate	1.6 Å	5NUB

## 2 Experimental design, materials and methods

The data listed above were collected on two beamlines at the European Synchrotron Radiation Facility (ESRF) in Grenoble. The diffraction data were collected from crystals cooled to 100 K after brief immersion into a mixture of 80% mother liquor and 20% glycerol. The data were indexed, integrated and reduced using the programs MOSFLM [5] and Scala [6]. The processed data were converted to structure factors using the program TRUNCATE from the CCP4 suite [7]. More details on these data are summarized in Table 1 of the article “Human plasma retinol-binding protein (RBP4) is also a fatty acid-binding protein” [4].

**Table 1:** Main differences in holo RBP4 in the 2.5 and 1.5 Å resolution models.

**RBP4 residue**	**Atom**	**Δ-B (Å**^**3**^**)**	**Δ−XYZ (Å)**	**1.5 Å electron density map of the side chain**
**Arg 10**	**CZ**	**33.9**	**4.43**	**Good**
**Glu 13**	**OE2**	**24.8**	**6.03**	**Poor**
**Glu 49**	**OE2**	**24.5**	**5.03**	**Good**
**Lys 58**	**CE**	**32.6**	**2.72**	**Absent**
**Leu 63**	**CD1**	**39.9**	**3.12**	**Poor**
**Leu 64**	**CD1**	**48.0**	**5.93**	**Absent**
**Asn 65**	**OD1**	**19.4**	**1.50**	**Absent**
**Asn 66**	**ND2**	**28.8**	**2.56**	**Absent**
**Trp 67**	**CH2**	**46.2**	**4.47**	**Poor**
**Asp 68**	**O**	**8.49**	**1.54**	**Good**
**Val 69**	**CG2**	**49.2**	**2.56**	**Poor**
**Cys 70**	**SG**	**19.7**	**2.33**	**Excellent**
**Asp 72**	**OD2**	**49.9**	**1.58**	**Excellent**
**Phe 77**	**CE2**	**1.85**	**2.46**	**Excellent**
**Glu 81**	**OE1**	**26.1**	**4.04**	**Absent**
**Phe 86**	**CE1**	**1.21**	**2.69**	**Excellent**
**Lys 87**	**CE**	**3.54**	**2.86**	**Good up to CD**
**Phe 96**	**CD2**	**10.7**	**2.45**	**Good**
**Gln 98**	**OE1**	**1.05**	**2.33**	**Excellent**
**Lys 99**	**CE**	**5.04**	**2.56**	**Absent**
**Asp 112**	**OD1**	**15.9**	**3.61**	**Excellent**
**Tyr 114**	**CD2**	**14.1**	**2.64**	**Excellent**
**Arg 121**	**NH2**	**2.9**	**2.53**	**Excellent**
**Leu 125**	**CD2**	**8.6**	**2.62**	**Excellent**
**Tyr 133**	**CE1**	**2.0**	**2.50**	**Excellent**
**Glu 147**	**OE2**	**47.0**	**6.85**	**Absent**
**Gln 149**	**OE1**	**6.68**	**4.11**	**Excellent**
**Lys 150**	**CE**	**30.4**	**3.10**	**Absent**
**Gln 154**	**OE1**	**10.7**	**3.09**	**Absent**
**Glu 157**	**OE2**	**41.1**	**2.59**	**Excellent**
**Arg 163**	**NH2**	**26.1**	**7.18**	**Good**
**Arg 166**	**NH2**	**40.6**	**7.95**	**Absent**
**Leu 167**	**CD1**	**4.9**	**2.65**	**Excellent**
**Tyr 173**	**CE2**	**6.9**	**3.85**	**Absent**

In Table 1 we summarize the largest differences between the models of the same crystal form of RBP4 purified from plasma and solved to resolutions of 2.5 Å [3] and 1.5 Å [4] respectively (PDB accession codes 1BRP and 5NU7). The first column of the table identifies the amino acid and the second the atom where the largest difference was found while the last column gives a description of the electron density quality in the two maps. Third and fourth column record the differences in B factors and the distance between the two positions measured in Angstroms.

In a similar manner, Table 2 compares the models of the same crystal form of RBP4 purified from plasma, not bound to retinol and solved to a resolution of 2.5 Å [3] and 2.0 Å [4].

**Table 2:** Main differences in non-fluorescent RBP4 in the 2.5 and 2.0 Å resolution models.

**RBP4 residue**	**Atom**	**Δ-B (Å**^**3**^**)**	**Δ-XYZ (Å)**	**2.0 Å electron density map of the side chain**
**Arg 10**	**CZ**	**39.1**	**4.60**	**Absent**
**Glu 13**	**OE1**	**48.6**	**4.85**	**Absent**
**Glu 44**	**OE1**	**71.9**	**3.02**	**Excellent**
**Phe 45**	**CE1**	**12.6**	**2.89**	**Excellent**
**Glu 49**	**OE2**	**31.7**	**4.61**	**Absent**
**Lys 58**	**NZ**	**39.9**	**3.00**	**Absent**
**Val 61**	**CG1**	**28.4**	**2.27**	**Good**
**Arg 62**	**NH1**	**29.3**	**2.71**	**Absent**
**Leu 64**	**CD2**	**9.8**	**6.25**	**Absent**
**Asn 65**	**OD1**	**19.6**	**2.21**	**Absent**
**Asn 66**	**O**	**23.6**	**2.63**	**Absent**
**Trp 67**	**CH2**	**4.5**	**4.58**	**Absent**
**Asp 68**	**OD2**	**30.6**	**1.70**	**Poor**
**Val 69**	**CG1**	**5.8**	**1.99**	**Poor**
**Cys 70**	**SG**	**32.1**	**2.08**	**Good**
**Phe 86**	**CE1**	**6.0**	**2.88**	**Excellent**
**Lys 87**	**CE**	**58.5**	**3.13**	**Good up to CD**
**Lys 99**	**NZ**	**12.2**	**3.67**	**Absent**
**Asp 112**	**OD2**	**5.4**	**2.05**	**Good**
**Arg 121**	**NH2**	**15.0**	**2.87**	**Excellent**
**Arg 139**	**NH1**	**29.4**	**2.67**	**Excellent**
**Glu 147**	**OE1**	**90.7**	**4.80**	**Absent**
**Lys 150**	**NZ**	**18.4**	**3.44**	**Absent**
**Ile 151**	**CD1**	**29.6**	**4.45**	**Excellent**
**Arg 153**	**NH1**	**6.0**	**2.48**	**Excellent**
**Gln 154**	**OE1**	**38.2**	**3.04**	**Absent**
**Arg 155**	**NH2**	**5.0**	**1.32**	**Good**
**Gln 156**	**NE2**	**14.2**	**2.66**	**Excellent**
**Glu 157**	**OE2**	**50.7**	**2.68**	**Good**
**Arg 163**	**NH2**	**16.5**	**6.64**	**Good**
**Gln 164**	**NE2**	**25.7**	**2.85**	**Excellent**
**Leu 167**	**CD2**	**15.6**	**2.39**	**Good**
**Tyr 173**	**CE2**	**16.3**	**3.83**	**Poor**
**Cys 174**	**O**	**3.3**	**1.46**	**Poor**

Table 3 analyses the differences between RBP4 purified from plasma and bound to palmitic acid and retinol.

**Table 3:** Main differences between non-fluorescent and holo RBP4 in the 2.0 and 1.5 Å resolution models (crystal forms 1 & 4 in Table 1 of reference [4]).

**RBP4 residue**	**Atom**	**Δ-B (Å**^**3**^**)**	**Δ-XYZ (Å)**	**Electron density map of the side chain, form 1**	**Electron density map of the side chain, form 4**
**Lys 29**	**NZ**	**14.5**	**4.56**	**Excellent**	**Excellent**
**Gly 34**	**O**	**3.1**	**1.91**	**Excellent**	**Excellent**
**Leu 35**	**CD2**	**10.4**	**9.53**	**Excellent**	**Good**
**Phe 36**	**CZ**	**9.1**	**9.18**	**Excellent**	**Excellent**
**Gln 52**	**OE1**	**26.8**	**2.34**	**Good**	**Good**
**Val 61**	**CG1**	**10.0**	**2.89**	**Good**	**Good**
**Tyr 114**	**CD1**	**8.7**	**2.39**	**Excellent**	**Excellent**
**Gln 149**	**OE1**	**5.76**	**4.48**	**Excellent**	**Excellent**
**Lys 150**	**NZ**	**14.6**	**2.20**	**Absent**	**Absent**
**Arg 153**	**NH2**	**2.0**	**1.68**	**Excellent**	**Excellent**
**Glu 157**	**OE2**	**31.2**	**3.4**	**Good**	**Excellent**
**Arg 166**	**NH2**	**31.4**	**8.95**	**Excellent**	**Absent**
**Val 169**	**CG1**	**16.8**	**2.44**	**Good**	**Good**

**Fig. 1:**
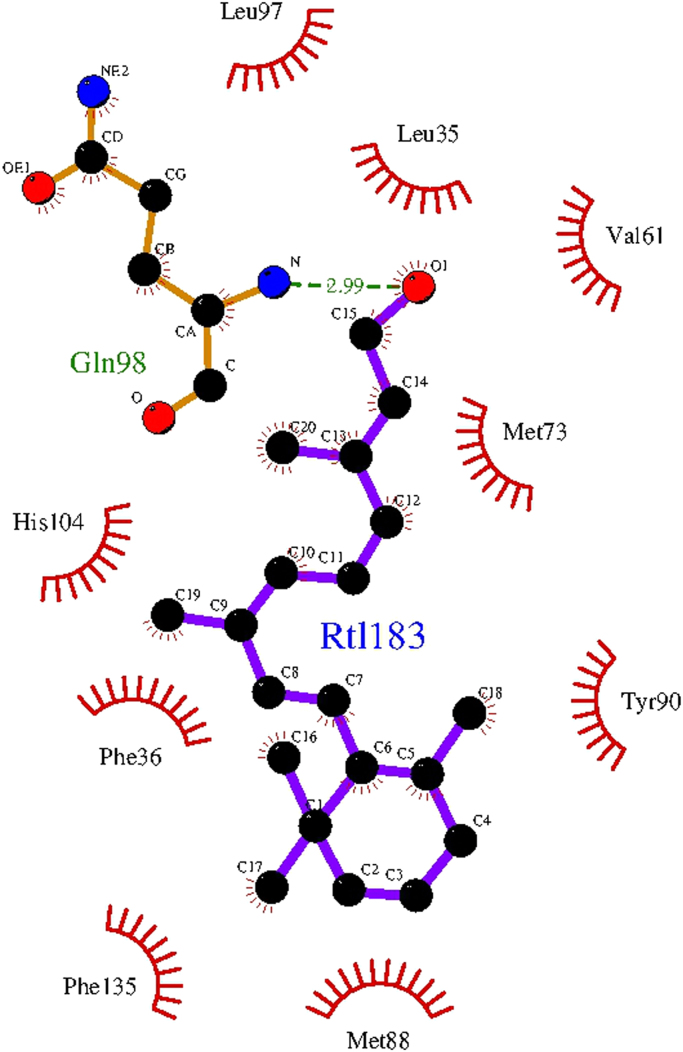
Gln^98^ and other side chains participating in the specific contacts of RBP4 with retinol. A hydrogen bond is indicated with green broken lines, whereas the amino acids that make hydrophobic contacts are only indicated but not represented as ball and stick models.

**Fig. 2:**
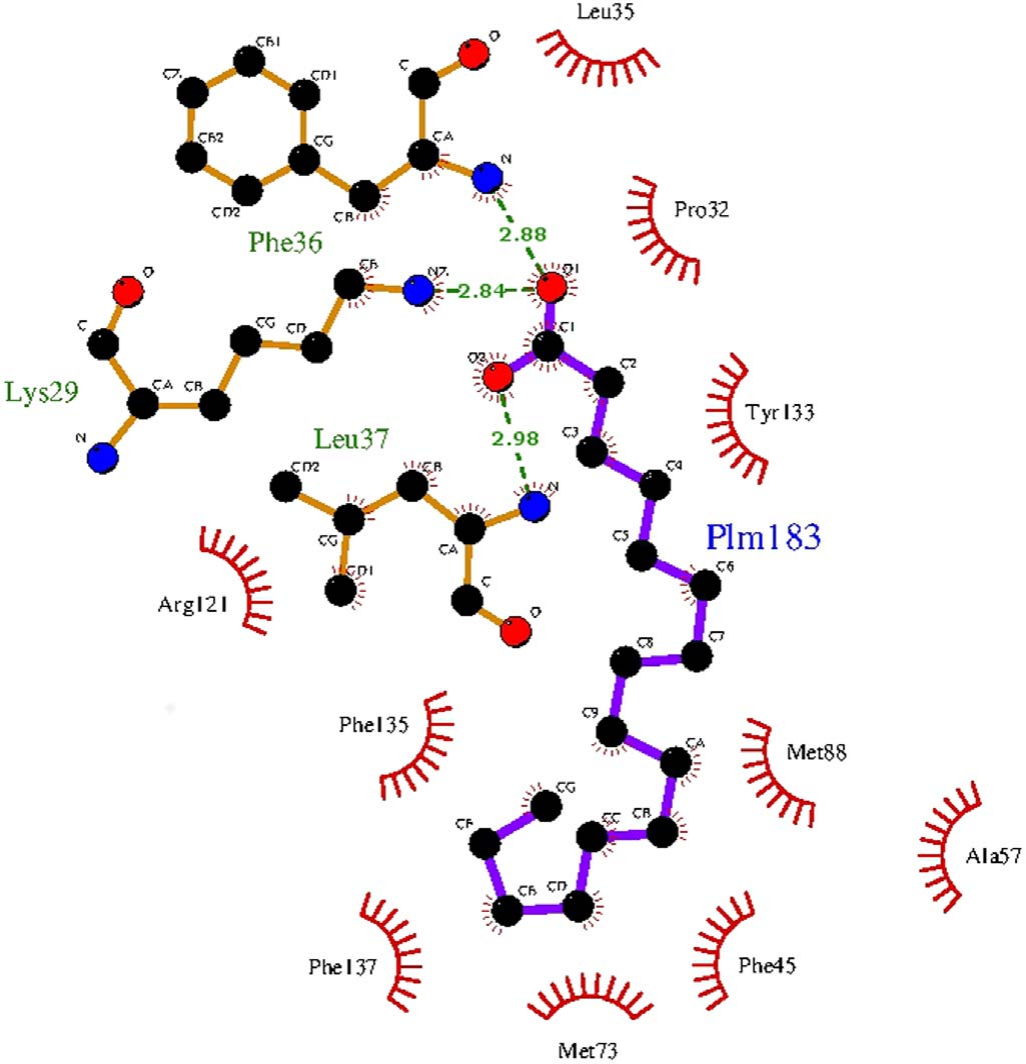
Interaction of Lys^29^, Phe^36^ and Leu^37^ and other side chains participating in the specific contacts of RBP4 with palmitic acid. Hydrogen bonds are indicated with green broken lines, whereas the amino acids that make hydrophobic contacts are only indicated but not represented as ball and stick models.

The shortest distances between RBP4 residues and three ligands, palmitate (Table 4), laurate (Table 5) and retinol (Table 6) are listed in the three tables. The main interactions between RBP4 and retinol and palmitate are represented in Fig. 1, Fig. 2 respectively.

**Table 4:** RBP4 residues in contact with palmitate in Crystal form 6.

Only the shortest distance per residue has been included in the table. The residues in yellow are in contact with all the ligands

**Table 5:** RBP4 residues in contact with laurate in Crystal form 8.

Only the shortest distance per residue has been included in the table. The residues in yellow are in contact with all the ligands

**Table 6:** RBP4 residues in contact with retinol in Crystal form 4.

Only the shortest distance per residue has been included in the table. The residues in yellow are in contact with all the ligands

Table 7 lists the ligand binding cavities of the two RBP4 populations analyzed, i.e bound to retinol and to fatty acids calculated from the coordinates of the models using the program CASTp [8].

**Table 7:** Comparison of the ligand-binding cavity volumes.

**Sample origin**	**Crystal form****[4]**	**Ligand**	**Resolution**	**Cavity Volume (Å**^**3**^**) CASTp**
**Plasma**	**1**	**Palmitate**	**2.00 Å**	**696.7**
**Urine**	**2**	**Palmitate**	**1.50 Å**	**659.7**
**Amniotic Fluid**	**3**	**Palmitate**	**1.68 Å**	**682.2**
**Plasma**	**4**	**Retinol**	**1.50 Å**	**789.3**
**Urine**	**5**	**Palmitate**	**1.59 Å**	**662.8**
**Amniotic Fluid**	**6**	**Palmitate**	**1.50 Å**	**666.7**
**Urine**	**7**	**Laurate**	**1.60 Å**	**657.9**
**Amniotic Fluid**	**8**	**Laurate**	**1.60 Å**	**657.5**

The cavity volume computations were done with the program CASTp [8]
