# Supplementary material for: High resolution crystal structure data of human plasma retinol-binding protein (RBP4) bound to retinol and fatty acids
Source: Data Brief. 2018 Mar 29;18:1073–81. doi: 10.1016/j.dib.2018.03.112 (PMC5996608; doi:10.1016/j.dib.2018.03.112)
Supplement: Supplementary file 1 — Supplementary material [file mmc1.docx]

Declarations of Interest

None.
